# Supplementary material for: The SARS-CoV-2 Spike Protein Receptor-Binding Domain Expressed in Rice Callus Features a Homogeneous Mix of Complex-Type Glycans
Source: Int J Mol Sci. 2024 Apr 18;25(8):4466. doi: 10.3390/ijms25084466 (PMC11050186; doi:10.3390/ijms25084466)
Supplement: Supplementary file 1 [file ijms-25-04466-s001.zip › ijms-2938934-supplementary.pdf]

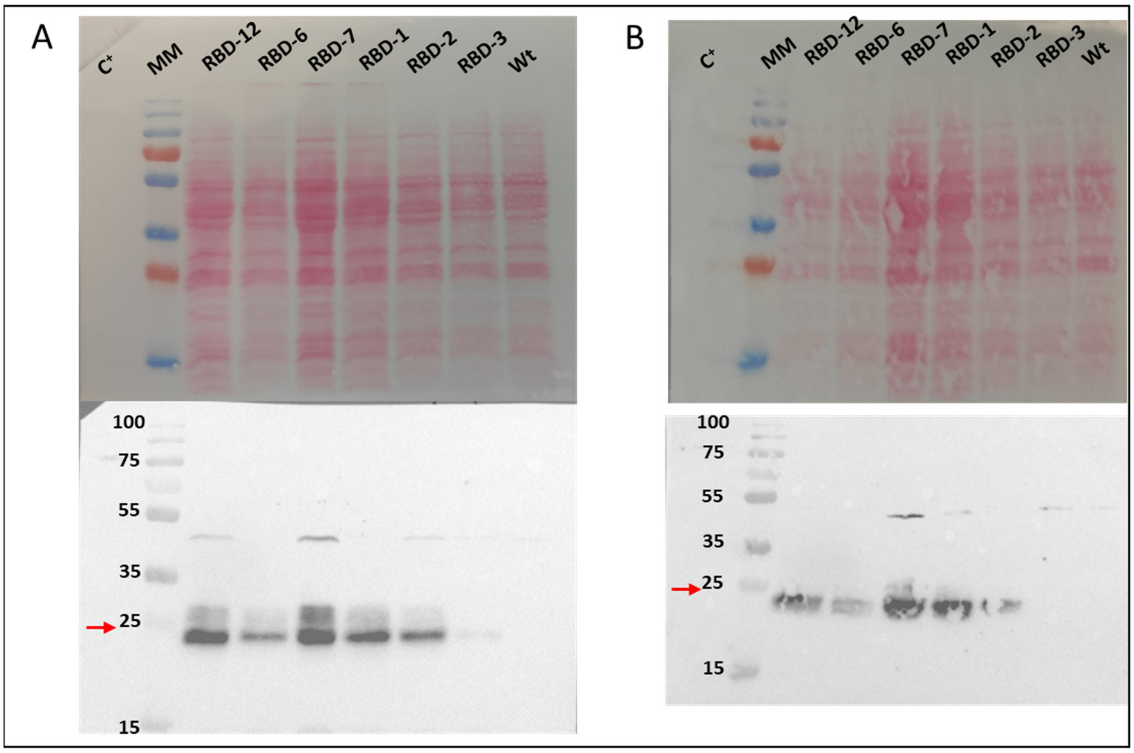

Supplementary Figure S1. Western blot of protein extracts from pUbi-RBD lines. Primary antibodies against (A) the His_6 tag and (B) the RBD were used to detect the recombinant protein. Upper panels show Ponceau S staining for total protein. Lower panels show corresponding western blots. C^+ = pure RBD positive control. MM = molecular marker. WT = total protein extract from wild type callus (negative control). Other lanes represent independent RBD callus lines each containing 40 µg of protein. Red arrows indicate the expected size of the recombinant RBD.

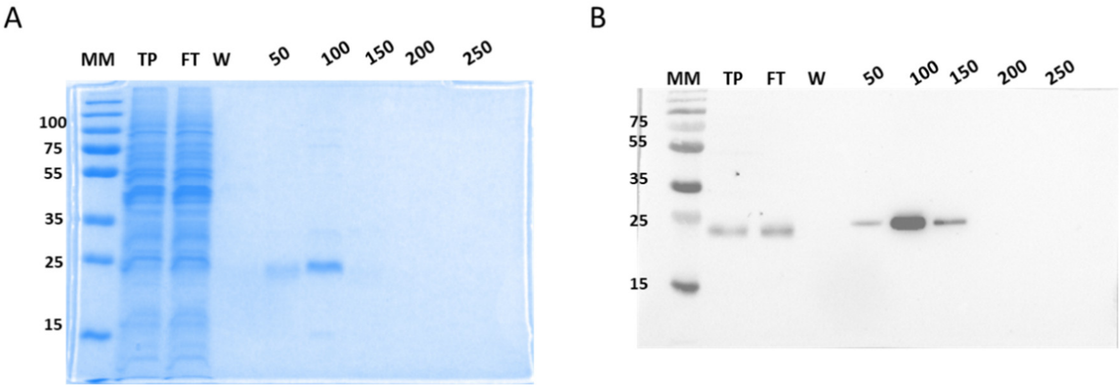

Supplementary Figure S2. Analysis of RBD purification fractions. (A) SDS-PAGE followed by staining with Coomassie Brilliant Blue and (B) western blot analysis of the total protein (TP) before purification, flow through (FT), washes (W) and various elution fractions at different imidazole concentrations: 50, 100, 150, 200 and 250 mM. A primary anti-RBD antibody was used to detect the recombinant RBD protein.

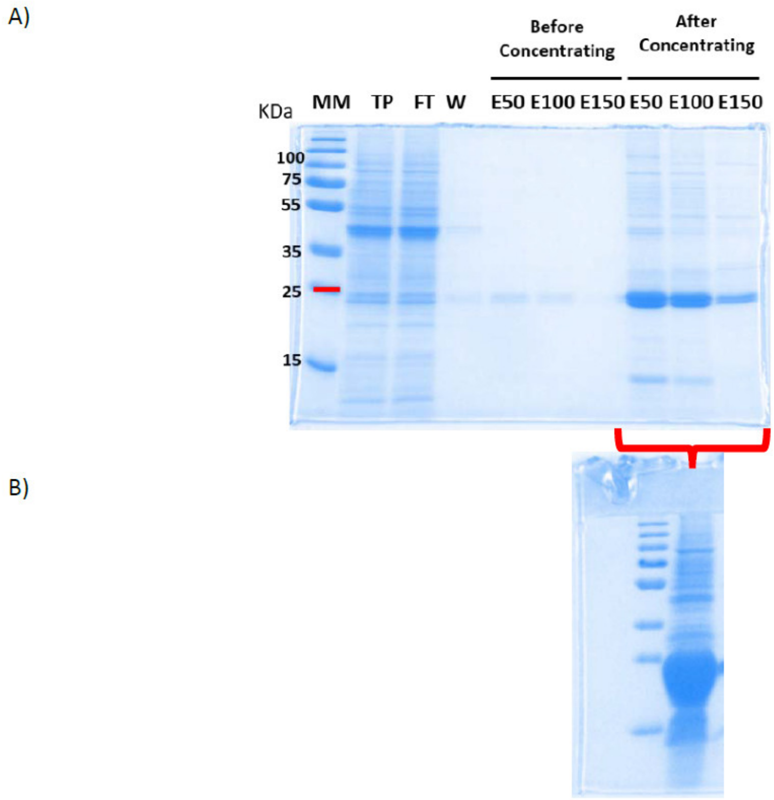

Supplementary Figure S3. SDS-PAGE analysis and Coomassie staining of different fractions collected during purification. (A) We analyzed the total protein (TP) before purification, flow through (FT), washes (W) and various elution fractions at different imidazole concentrations: 50, 100 and 150 mM (E50, E100, E150) before and after concentration. (B) The elution fractions were pooled for subsequent analysis. MM = molecular markers.

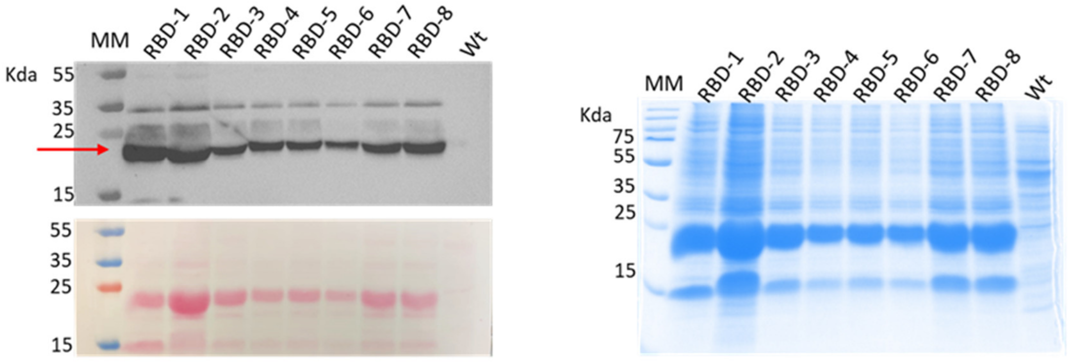

(A) **(B)**

Supplementary Figure S4. Analysis of different batches of RBD. (A) Western blot and Ponceau S staining of 40-µg samples detected with a primary antibody against the His_6 tag. (B) SDS-PAGE followed by Coomassie staining of the same samples. MM = molecular marker. WT = negative control wild-type callus.
